# Evaluation of the Effect of *Hypericum triquetrifolium* Turra on Memory Impairment Induced by Chronic Psychosocial Stress in Rats: Role of BDNF

**DOI:** 10.2147/DDDT.S278153

**Published:** 2020-12-01

**Authors:** Karem H Alzoubi, Laila Abdel-Hafiz, Omar F Khabour, Tamam El-Elimat, Mohammad A Alzubi, Feras Q Alali

**Affiliations:** 1 Department of Clinical Pharmacy, Faculty of Pharmacy, Jordan University of Science and Technology, Irbid 22110, Jordan; 2 Institute of Anatomy II, Medical Faculty, Heinrich Heine Universität, Düsseldorf, Germany; 3 Department of Medicinal Chemistry and Pharmacognosy, Faculty of Pharmacy, Jordan University of Science and Technology, Irbid 22110, Jordan; 4 Department of Medical Laboratory Sciences, Jordan University of Science and Technology, Irbid 22110, Jordan; 5 Integrative Life Sciences Doctoral Program, Department of Pathology, Virginia Commonwealth University, Richmond, VA, USA; 6 College of Pharmacy, QU Health, Qatar University, Doha Qatar; 7 Biomedical and Pharmaceutical Research Unit, QU Health, Qatar University, Doha, Qatar

**Keywords:** *Hypericum triquetrifolium*, methanolic extract, stress, learning, memory, hippocampus, BDNF

## Abstract

**Background:**

Chronic psychosocial stress impairs memory function and leads to a depression-like phenotype induced by a persistent status of oxidative stress. *Hypericum perforatum* L. (St. John’s wort) is widely used to relieve symptoms of anxiety and depression; however, its long-term use is associated with adverse effects. *Hypericum triquetrifolium* Turra is closely related to *H. perforatum*. Both plants belong to *Hypericaceae* family and share many biologically active compounds. Previous work by our group showed that methanolic extracts of *H. triquetrifolium* have potent antioxidant activity as well as high hypericin content, a component that proved to have stress-relieving and antidepressant effects by other studies. Therefore, we hypothesized that *H. triquetrifolium* would reduce stress-induced cognitive impairment in a rat model of chronic stress.

**Objective:**

To determine whether chronic treatment with *H. triquetrifolium* protects against stress-associated memory deficits and to investigate a possible mechanism.

**Methods:**

The radial arm water maze (RAWM) was used to test learning and memory in rats exposed to daily stress using the resident–intruder paradigm. Stressed and unstressed rats received chronic *H. triquetrifolium* or vehicle. We also measured levels of brain-derived neurotrophic factor (BDNF) in the hippocampus, cortex and cerebellum.

**Results:**

Neither chronic stress nor chronic *H. triquetrifolium* administration affected performance during acquisition. However, memory tests in the RAWM showed that chronic stress impaired different post-encoding memory stages. *H. triquetrifolium* prevented this impairment. Furthermore, hippocampal BDNF levels were markedly lower in stressed animals than in unstressed animals, and chronic administration of *H triquetrifolium* chronic administration protected against this reduction. No significant difference was observed in the effects of chronic stress and/or *H. triquetrifolium* treatment on BDNF levels in the cerebellum and cortex.

**Conclusion:**

*H. triquetrifolium* extract can oppose stress-associated hippocampus-dependent memory deficits in a mechanism that may involve BDNF in the hippocampus.

## Introduction

In biological terms, stress can be defined as any condition that interrupts the equilibrium between an organism and its living environment and causes the release of stress mediators, including glucocorticoid hormones.^1^,^2^ Stress triggers a sequence of events in the brain and peripheral nervous system to help the organism cope with and adapt to challenging situations.^3^ However, when stress is maintained for an extended period, most physiological systems will be negatively affected.^4^

The complicated relationship between stress and cognition has been a major focus of research over the past century. Studies have shown that stress can variably impair, enhance, or have no effect on learning and memory processes in rodents, depending on the kind of stressor and its duration, the task used to evaluate cognition, and the age, species and strain of the animal.^5–8^

However, most studies agreed that prolonged stress negatively affects brain functions and impacts nearly every brain region.^9^ Chronic stress impairs cognition in several aspects such as memory acquisition, consolidation and recall.^10^ Most studies have focused on the relationship between prolonged stress and spatial ability. Decades of research have identified the hippocampus as an essential part for spatial ability, and showed that chronic stress influences hippocampal function, thus affecting spatial learning and memory.^11^ The structure of the hippocampus was found to be particularly sensitive to stress because of its high density of glucocorticoid receptors.^1^ Under situations of prolonged stress, the hippocampus undergoes disturbances in neurogenesis,^12^ as well as neuronal atrophy,^13^,^14^ leading to a corresponding reduction in the total number of neurons and their ramifications.^15^,^16^

Stress also has a crucial role in the etiology of anxiety and mood disorders such as depression.^17^,^18^ In rats, chronic stress leads to a depression-like phenotype induced by a persistent state of oxidative stress,^19^ which causes oxidative damage, specifically in the hippocampus.^20^ Many studies have shown that chronic stress and depression can influence the hippocampus in a highly dynamic manner. For instance, depression shrinks hippocampal volume,^21^ and causes deficits in hippocampus-dependent declarative memory.^22^ At the molecular level, depression is associated with a decline in the expression of brain-derived neurotrophic factor (BDNF).^23^ Moreover, reduced hippocampal plasticity in depressed individuals supports the literature on spatial ability and dendritic structure in the hippocampus following prolonged exposure to the stress in rodents.^24^ Together, this evidence demonstrates that chronic stress and depression are closely associated, and suggests that putative antidepressant agents, such as *H. perforatum*, should be investigated as therapeutic options to improve the negative outcomes of chronic exposure to stress.

BDNF plays a crucial role in synaptic plasticity and neuronal survival.^25^ Its neurotrophic action has been implicated in its improvement of normal,^26^ and impaired memory function.^27^ BDNF levels decline as well during chronic stress situations.^28^

The resident–intruder psychosocial stress paradigm is an animal model that produces stress by continuously disrupting the established social hierarchy.^29–31^ This method elevates serum corticosterone levels,^32^ and blood pressure,^33^ proving its validity as a chronic stress induction model. Chronic social stress also induces depression-like behaviors.^34^ Therefore, exposing rats to long-term social stress is expected to lead not only to deficits in spatial ability, but also to a depression-like phenotype.^35^

*H. perforatum* (St. John’s wort) is a commercially available medicinal plant used to relieve symptoms of depression.^36^ Studies using various behavioral learning and memory paradigms indicate it has nootropic activity,^37^,^38^ but further investigations are required to confirm this. *H. perforatum* extract also enhanced BDNF expression in a human-derived cell line.^39^ However, several clinical studies revealed that its long-term administration resulted in several side effects such as anxiety, restlessness, insomnia, and gastrointestinal disorders including diarrhea.^40–42^ One recent study in rats showed that long-term treatment with *H. perforatum* led to impairments in short- and long-term memory and decreased hippocampal BDNF levels.^43^ Therefore, a safer alternative to *H. perforatum* is needed that improves or has no effect on memory function under unstressed conditions, while counteracting the deleterious effect of chronic stress on spatial working and reference memory. Such an agent would provide us with an alternative treatment to be used daily to help us cope with the repeated, persistent stresses of everyday life. *H. triquetrifolium* also gained considerable scientific interest in the last years, due to its richness of a variety of bioactive compounds that make *H. triquetrifolium* to be a medicinal herb with a wide range of medicinal applications.^44^ *H. triquetrifolium* grows wild in Jordan,^45^ and it shares many active compounds with *H. perforatum*, but the plants display considerable phytochemical diversity.^46^ Generally, the therapeutic activity observed in medicinal plants is not attributed to a single active compound, and in most cases, extracts show activity based on the synergistic or antagonistic effects of their different components. Therefore, we used a holistic approach to investigating *H. triquetrifolium* by testing the effects of the methanolic extract, which contains many biologically active components and in preliminary work by our group, showed potent antioxidant activity.^46–48^

Despite the well-studied effect of *H. perforatum* on cognitive functions, *H. triquetrifolium* was poorly explored. For this purpose, *H. triquetrifolium* aerial parts were collected from the wild nature in Jordan, dried and subsequently extracted with different solvents to get the methanolic extract in order to evaluate its possible attenuating influence on stress-induced learning and memory impairment. Here, we report the first investigation of how *H. triquetrifolium* Turra, a closely related species to *H. perforatum*, affects cognitive processes and cognitive biomarkers in the brain. To the best of our knowledge, this is the first time to link the impact of *H. triquetrifolium* extract to learning and memory, *H. triquetrifolium* extract impact on stress-associated memory impairment, and chronic psychosocial stress to different forms of consolidation processes. We investigated the impact of chronic stress on different stages of hippocampus-dependent memory processing and tested the putative memory-enhancing properties of *H. triquetrifolium* methanolic extract. We then purified the methanolic extract and identified some of its active ingredients. Finally, we investigated the possible underlying mechanisms by which *H. triquetrifolium* exerts its effects by investigating BDNF levels in multiple brain areas, especially in the hippocampus, due to its well-known role in spatial learning and memory.^49^

## Methods

### Plant Collection

The aerial parts of *H. triquetrifolium* Turra were collected during the flowering stage in July from Al-Mafraq and the campus of Jordan University of Science and Technology (JUST), Irbid, Jordan. The collected plant material was identified by Dr. Mohammad Gharaibeh, a plant taxonomist at the Department of Natural Resources and Environment, Faculty of Agriculture, JUST. A voucher specimen (PHS-120) was deposited in the herbarium of the Faculty of Pharmacy, JUST, Irbid, Jordan. The raw material was cleaned and air-dried at room temperature, away from direct sunlight. The dried plant material was then ground to a fine powder using a laboratory mill (RetschMühle, Retsch GmbH, Haan, Germany) and stored at room temperature (22–23°C) in paper bags protected from light and humidity until required for analysis.

### Phytochemical Analysis

**Scheme 1:**
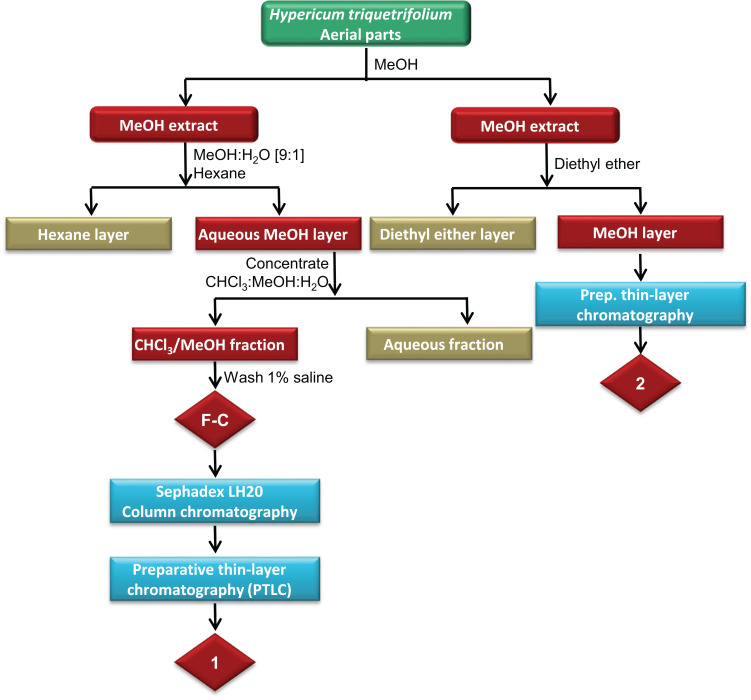
Extraction and fractionation scheme of the methanolic extract of the aerial parts of *H. triquetrifolium.*

The powdered plant material (1.1 kg) was extracted exhaustively for 3 h using methanol in a Soxhlet extractor. The solvent was evaporated to dryness under reduced pressure using a rotary evaporator (RE 200, Bibby Steriline Ltd., Stone, UK) to yield 237 g of the MeOH extract. The MeOH fraction was dissolved in 500 mL of MeOH:H_2_O [9:1] and partitioned with 1 L hexane. The aqueous methanolic layer (Fraction A) was then concentrated and partitioned between CHCl_3_:MeOH (4:1) and water (1:1). After this, the chloroform/methanolic fraction (Fraction B) was washed with 1% saline to yield 19.9 g extract (Fraction C), of which about 1 g was further purified using normal phase successive Sephadex LH20 column chromatography and preparative thin-layer chromatography (PTLC) (Scheme 1).

About 40 g of Sephadex (bead size 25–100 μm, Sigma-Aldrich, St Louis, MO, USA) was swollen for 7 h in 100 mL HPLC-grade ethanol to obtain a final bed volume of 160 mL. Isocratic elution with 100% HPLC-grade ethanol was performed. The subtractions obtained were then purified using successive Sephadex LH20 column chromatography and PTLC. For PTLC, the following mobile phase system was used: ethyl acetate: glacial acetic acid: formic acid: water, at a ratio of 10:1.1:1.1:2.6 to yield compound 1 (Scheme 1).

Compound 2 was isolated from the methanolic extract of *H. triquetrifolium* based on the method of^50^). In brief, about 57 g of the dried methanolic extract was washed with 200 mL of diethyl ether five times at room temperature until no fluorescent pink color was observed in the ether phase in the presence of ultraviolet radiation. Successive PTLC was used for the isolation of 2 by dissolving the carotenoid pigment-free extract in ethanol. The mobile phase solvent system used was ethyl acetate: glacial acetic acid: formic acid: water, at a ratio of 10:1.1:1.1:2.6.

The chemical structures of the isolated compounds were elucidated using mass spectrometry (MS) and nuclear magnetic resonance (NMR) spectroscopy and by comparison with an authentic standard of known compounds. NMR analysis of the isolated compounds was conducted using a 400 MHz Bruker spectrometer. Mass spectra were obtained using an ion-trap mass spectrometer (Agilent, Palo Alto, CA, USA) equipped with an electrospray ionization source and an Agilent 100 series HPLC.

### Pharmacological Studies

#### Animals

Adult male Wistar rats (starting weight 150–250 g each) were used and housed in metal cages (2 to 5 animals per cage). The rats were housed up to five rats per cage under a 12 h light/dark cycle (light off 7AM) at 25°C, with free access to food and tap water. The study protocol was approved by the Institutional Animal Care and Use Committee of Jordan University of Science and Technology (Approval number: 74/2008). Work was in compliance with The Institutional Animal Care and Use Committee Guidebook, 2nd edition of 2002.^51^

**Figure 1:**
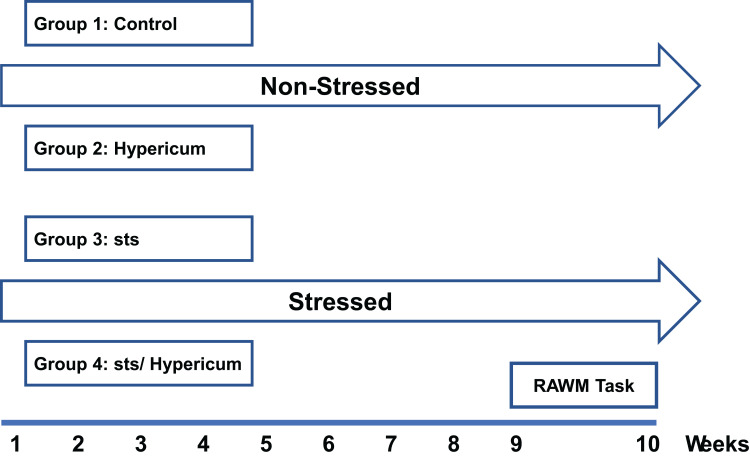
Experimental procedure for the different experimental groups. Animals were assigned to four groups at random: Control, Hypericum, sts and sts/Hypericum. The sts and sts/Hypericum groups were exposed to chronic daily psychosocial stress using the resident–intruder model for 9 weeks. H. triquetrifolium (50 mg/kg/day i.p.) was administered daily for 9 weeks in the Hypericum and sts/Hypericum groups. Control and sts groups received the vehicle without plant extract once daily for 9 weeks. Behavioral assessment of spatial working and reference memory commenced at week 10.

Animals were randomized to four groups of 12–15 animals each: Vehicle-treated group (control); *H. triquetrifolium* extract (Hypericum); chronic stress (sts); chronic stress + *H. triquetrifolium* (sts/Hypericum). The sts and sts/Hypericum groups were subjected to chronic psychosocial stress for 9 weeks using the resident–intruder model (detailed 2.3.2). While control and Hypericum groups were handled and transferred to the experimental room and returned to their home cage every day. During this experimental period, the Hypericum and sts/Hypericum groups received *H. triquetrifolium* (50 mg/kg/day i.p.) once daily, dose was selected based on memory-enhancing properties of *H.perforatum* extract on the conditioning of avoidance learning in rats (Klusa et al, 2001). Both *H. triquetrifolium* and *H. perforatum* belong to *Hypericaceae* family and share many of biologically active compounds.^46^ The control and sts groups received equivalent volumes of the vehicle (0.3% DMSO and 0.1 M NaOH at a ratio of 1:1) without plant extract, on the same schedule (Figure 1). All experimental protocols were conducted during the dark period in a dimly lit room.

#### Induction of Chronic Psychosocial Stress

Chronic stress was established in sts and sts/Hypericum using the intruder model as previously described (Alzoubi et al 2013 a, b). Briefly, two groups of animals, marked as stressed only “sts” and stressed- *H. perforatum* “sts/Hypericum”, were housed with the same cage mates for 2 weeks to establish a social hierarchy. Stress was then induced by swapping two rats (chosen pseudo-randomly) daily between those cages from each cage to the other for a period of 9 weeks. Thus, animals were forced to continually adapt to new stressful situations as daily swapping disrupts the social hierarchy among animals.^52^,^53^ This method was shown by our group to elevate serum corticosterone levels,^32^ and blood pressure,^33^ indicating that it induces chronic stress.

#### Radial Arm Water Maze (RAWM)

After 9 weeks of sts and/or Hypericum treatment, learning and memory performance in the RAWM was tested in all experimental groups. We used the same procedure for the spatial working memory version of RAWM as described by.^32^,^54^ The RAWM is a black circular water tank with six V-shaped stainless steel plates arranged to form a swimming field with an open central area and six arms filled with water (24 ± 1°C). A transparent escape platform was hidden/submerged 2 cm beneath the water surface at the far end of one of the swim arms (goal arm). Animals had to find this hidden platform. Briefly, there were four phases in the procedure: acquisition, followed by three memory tests.

To begin a trial, each animal was placed into the pool facing the wall at one of the five possible starting points or “start arm” randomly chosen (any arm other than the goal arm), and allowed to freely swim in the maze until it found the hidden platform. A correct choice scored when the animal swam into the goal arm, climbed onto the hidden escape platform and allowed to remain on it for 15 s. However, every time the animal entered incorrect arm, an error was counted. The number of errors made before the animal found the platform was recorded. The maximum time allowed to perform the task was 60 sec.

The RAWM phases were the learning phase (acquisition phase), followed by three memory tests. In the learning phase, each animal was tested by conducting 12 learning-trials (two sessions, each session consisted of 6 trials with 5 min rest in-between; after the first six trials). After completion of the acquisition sessions (the learning phase), the animal’s memory was tested at 30 min, 5 h, and 24 h time periods after ending the learning phase (1 trial per test session). The final memory test (24h test) was conducted just before the next acquisition session. The location of the platform was fixed in one arm “goal arm” for each day, but was relocated to another arm on the subsequent day in order to determine if the rat learned a problem-solving strategy and cognitive flexibility.

The training was continued for five consecutive days or until the animal reached days to criterion (DTC) in the last acquisition trial (trial number 12) and all memory tests (30 min, 5 h, and 24 h). The DTC is the number of days the animal took to make no errors (or zero error) in two consecutive days. All experiments were carried out during the animals’ active phase. Numerous extra-maze cues were pasted on the walls around the maze to serve as spatial cues including one white circle with diameter of 52 cm and one rectangle with black-white stripes with 50 x 43 cm dimensions. Groups were compared based on DTC, number of errors per acquisition trial, and number of errors per memory test. It is important to note that animals were time staggered into four stages, each stage had 3–4 animals from each experimental group, so that in each day only 15 animals needed to go through the RAWM paradigm.

### Biochemical Analysis

#### Brain Tissue Dissection

Twenty four hours after the last memory test brains were dissected over a frosted glass plate (4°C) containing glucose solution, placed on top of crushed ice. The hippocampus, cortex, and cerebellum were obtained from each brain. They were then moved into separate tubes that were immersed in liquid nitrogen and stored at −80ºC until analysis.

#### Determination of BDNF Levels

Brain tissues were homogenized in lysis buffer (137 mM NaCl, 20 mM Tris-HC1 pH 8, 0.1% NP-40, 10% glycerol, 0.5 mM Na_3_VO_4_, 1 mM phenylmethylsulfonyl fluoride, and protease inhibitor cocktail (Sigma-Aldrich)). Homogenates were centrifuged (14,000 × g for 5 min at 4°C) to remove insoluble material. Total protein concentrations were estimated using a Bradford assay kit (BioRAD, Hercules, CA, USA) according to the manufacturer’s instructions. To quantify BDNF protein, samples were diluted 1:4 in Dulbecco’s phosphate-buffered saline (0.2 g KC1, 8.0 g NaCl, 0.2 g KH_2_PO, 1.15 g Na_2_HPO, 100 mg MgCl_2_.6H_2_O and 133 mg CaCl_2_) and then BDNF protein was measured using an enzyme-linked immunosorbent assay (ELISA; Human/Mouse BDNF DuoSet kit, R&D Systems, Minneapolis, MN, USA) according to the manufacturer’s instructions. A BDNF standard curve (0–1000 pg/mL) was freshly prepared in a mixture of lysis buffer and Dulbecco’s phosphate-buffered saline (1:4). Incubation times and washes were as described in Adlard and Cotman, 2004. ELISA plates were read at 450 nm using an automated plate reader (Stat Fax 2100, Awareness Technology, Palm City, FL, USA).

### Statistical Analysis

The number of errors on acquisition in the RAWM were compared via two-way repeated measures ANOVAs followed by post test for multiple comparisons. The repeated measures factor was time. Whereas treatment and stress as between-subject factors. For DTC in the RAWM and the biochemical assays results, two-way ANOVA and Tukey’s post hoc test were used. The significance level was set as α ≤ 0.05. All analyses were performed in GraphPad Prism 6. Values all over the study were reported as mean ± SEM.

## Results

### Phytochemical Analysis

Fraction C of the methanolic extract of the aerial parts of *H. triquetrifolium* afforded the known flavonoid rutin (1) (obtained as a yellowish powder in 1.8% (wt/wt) yield, calculated based on the dry weight of the whole plant). The spectral data of the compound were in agreement with those for rutin standard as well as those reported in the literature.^55^,^56^

**Figure 2:**
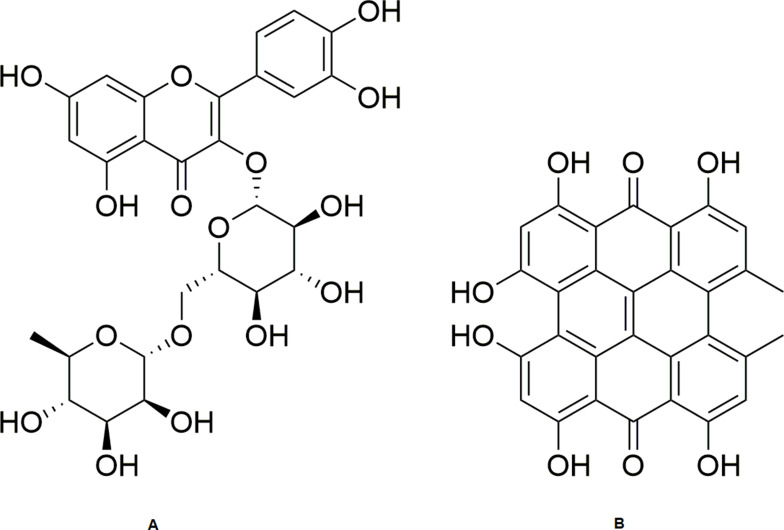
Structures of rutin (**A**) and hypericin (**B**).

Carotenoid-free methanolic extract of the aerial parts of *H. triquetrifolium* afforded the known naphthodianthrone hypericin (2) (Figure 2). The compound was obtained as a reddish powder in 0.417% (wt/wt) yield, calculated from the dry weight of the whole plant. The spectral data of the compound were in agreement with those for hypericin standard as well as those reported in the literature.^57^

### Evaluation of Plant Extract (*H. triquetrifolium*)

#### Combined Effect of *H. triquetrifolium* on Learning and Memory in Chronically Stressed Rats

Animals were trained on a new goal every day; thus, they all started with a high number of errors at the beginning of each training day. The number of errors declined as the number of training trials increased, indicating that the animals were learning the task effectively, by recalling the within-day platform location.

**Figure 3:**
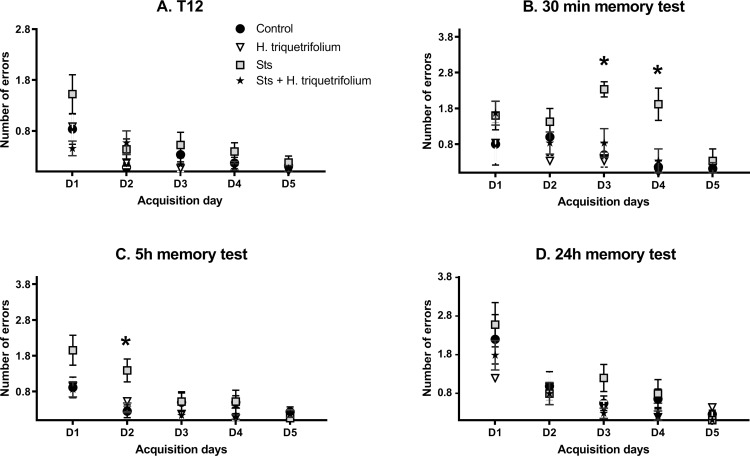
Performance in the radial arm water maze task (days 1–5). (**A**) The last acquisition trial (T12) represents the learning or acquisition phase, which was followed by (**B**) the 30 min, (**C**) 5 h and (**D**) 24 h memory tests. The sts group showed a significant performance deficit (more errors) in the 30 min and 5 h memory tests, which was reversed by chronic *H. triquetrifolium* administration. Values are means ± SEM (n = 12–15 rats per group); *p ≤ 0.05.

In the comparison of the number of errors committed at the end of each daily acquisition session (last acquisition trial, T12) between the sts and control groups, a significant main effect of day F_4196_ = 5.36 (p = 0.004) was found. However, neither the main effect of group nor the day × group interaction reached significance: day, F_1169_ = 3.626 (p > 0.05); group × day, F_4196_ = 0.45 (p > 0.05). When the Hypericum and sts/Hypericum groups were each compared to the control group, there was a significant main effect of day (F_4161_ = 4.081, p = 0.004) but not group (F_1161_ = 0.218; p > 0.05), with no group × day interaction (F_4161_ = 2.135; p > 0.05) (Figure 3, T12).

However, in the comparison of the average number of errors committed in each daily acquisition session (the means of 12 trials per day) between the sts and control groups, a significant main effect of day F_4164_ = 28.83 (p <0.001) and group (F_1164_ = 19.41; p < 0.001) was found, but not their interaction; group × day, F_4164_ = 1.602 (p > 0.05). Post hoc tests revealed significant differences in errors committed to reach the platform on acquisition days 1, 2 and 3 (p = 0.016, 0.0114 and 0.001, respectively), while day 4 revealed a near significant value (p= 0.0551). When the Hypericum and sts/Hypericum groups were each compared to the control group, there was a significant main effect of day (F_4161_ = 29.05, p < 0.001) but not group (F_1161_ = 3.197; p =0.076), with no group × day interaction (F_4161_ = 0.730; p > 0.05). These results indicate that chronic stress may have impaired learning process, and that this effect was prevented by chronic *H. triquetrifolium* administration.

In the short-term (30 min) memory test, the number of errors committed by animals in the sts group was higher than those in the other experimental groups. Results revealed a significant main effect of group (F_1,66_ = 17.33; p < 0.001) and day (F_4,66_ = 3.54; p = 0.011), but not their interaction (F_4,66_ = 2.137; p > 0.05). Post hoc tests revealed significant differences in errors committed to reach the platform on acquisition days 3 and 4 (p = 0.001 and 0.017, respectively). However, when the sts/Hypericum group was compared to the control group, a significant main effect of day (F_4,61_ = 4.57; p = 0.003) but not group (F_1,61_ = 1.533; p > 0.05) was found, as well as a group × day interaction (F_4,61_ = 0.729; p > 0.05) (Figure 3; 30 min test). Together, these results indicate that chronic stress impaired short-term memory, and that this effect was prevented by chronic *H. triquetrifolium* administration.

In the 5 h memory test, when comparing the sts and control groups, there was a significant main effect of group (F_1164_ = 4.64; p = 0.033) and day (F_4164_ = 5.212; p < 0.001) but no group × day interaction (F_4164_ = 2.186; p > 0.05) (Figure 3). Post hoc tests revealed significant differences in time to reach the platform location on acquisition day 2 (p = 0.019). Comparing the sts/Hypericum group with the control group revealed a main effect of day (F_4161_ = 4.781; p = 0.001) but not group (F_1161_ = 1.95; p > 0.05) and no group × day interaction (F_4161_ = 0.75; p > 0.05), indicating that chronic stress also induced long-term memory deficits in the 5 h memory test, and that this was prevented by chronic *H. triquetrifolium* administration. In the long-term (24 h) memory tests, no significant differences were detected in performance between any groups on any day (Figure 3). Notably, chronic *H. triquetrifolium* administration did not affect memory performance in unstressed animals in any of the memory tests, indicated by the comparable number of errors committed in the Hypericum and control groups (p > 0.05).

**Figure 4:**
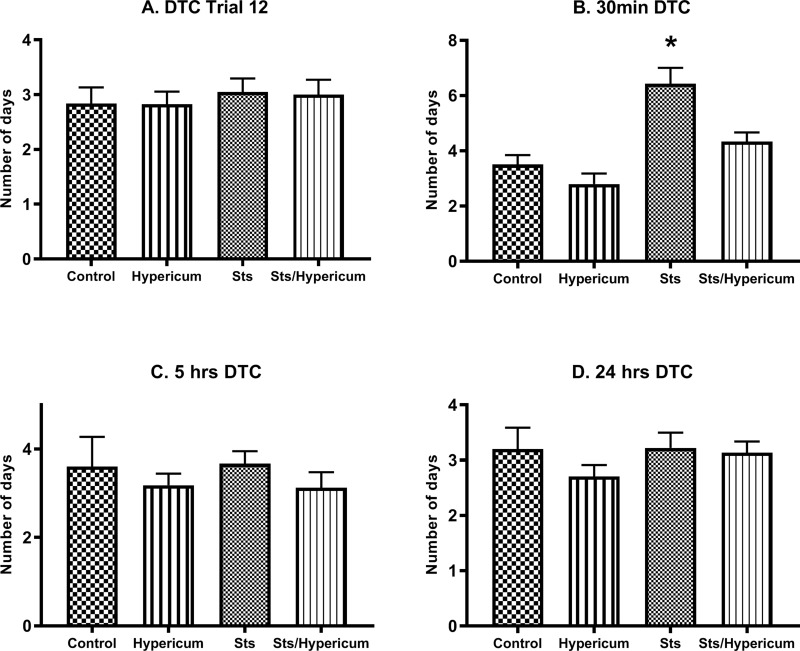
Memory function measured by days to criterion (DTC) (no errors in two consecutive days) for (**A**) trials 12, which is the last trail in the acquisition (learning) phase, (**B**) 30 min short-term memory test, (**C**) 5 hrs, and (**D**) 24 hrs long-term memory test. The sts group showed a significantly higher DTC than the control group, indicating that chronic stress impaired 30 min short-term memory. This effect was reduced by chronic *H. triquetrifolium* administration. Values are mean ± SEM (n = 12–15 rats per group); *p ≤ 0.05.

Two-way ANOVA revealed no difference in DTC between groups at T12 (F_3,67_ = 0.184; p > 0.05), 5 h (F_3,40_ = 0.611; p > 0.05) and 24 h (F_3,63_ = 0.921; p > 0.05) (Figure 4A, C and D). In contrast, there was a significant difference in DTC between groups in the 30 min memory test (F_3,24_ = 13.01; p < 0.001) (Figure 4B). A post hoc independent samples Tukey’s test showed that rats in the sts group needed significantly more days to reach criterion than those in the control, Hypericum, and sts/Hypericum groups for the 30 min memory test (p < 0.05) (Figure 4B), indicating impaired short-term memory in the sts group that was prevented in the sts/Hypericum group.

#### Combined Effect of *H. triquetrifolium* on Hippocampal BDNF Levels in Chronically Stressed Rats

**Figure 5:**
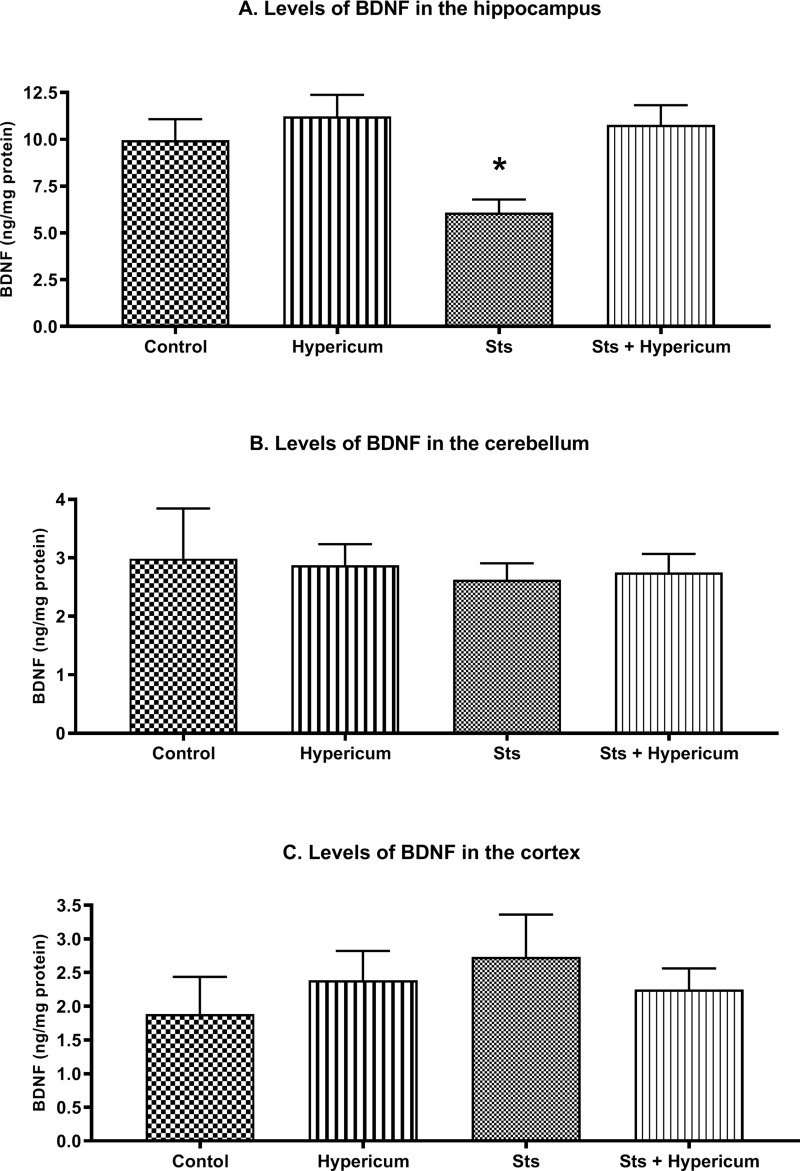
BDNF levels in the hippocampus, cerebellum, and cortex of rats in the Control, Hypericum, sts and sts/Hypericum groups. (**A**) Chronic stress reduced the levels of BDNF in the hippocampus; this was reversed by *H. triquetrifolium*. (**B, C**) No differences in BDNF levels were observed between groups in the cerebellum (**B**) or cortex (**C**). Values are means ± SEM (n = 12–15 rats per group); *p ≤ 0.05.

Two-way ANOVA revealed a significant difference in BDNF levels in the hippocampus between groups (F_3,50_ = 5.982; p = 0.001). Accordingly, a post hoc Tukey’s test for independent samples showed significantly lower BDNF levels in chronically stressed rats than in the control, Hypericum and sts/Hypericum groups (p > 0.05) (Figure 5A). Moreover, chronic administration of *H. triquetrifolium* extract normalized the reduction in BDNF levels and was comparable to the control group (p > 0.05). No significant difference was observed in the effects of chronic stress and/or *H. triquetrifolium* treatment on BDNF levels in the cerebellum and cortex (Figure 5B and C).

## Discussion

In the present study, we examined the influence of chronic stress on hippocampus-dependent spatial learning and memory, and investigated whether the effects could be prevented by chronic administration of a methanolic extract of *H. triquetrifolium* Turra. Behavioural effects were tested in the RAWM, a modified version of a standard task commonly used to evaluate hippocampus-dependent spatial learning and memory.^58^,^59^ Chronic administration of *H. triquetrifolium* prevented chronic psychosocial stress-induced impairments in spatial learning and memory performance and normalized the stress-induced reduction in hippocampal BDNF levels.

BDNF links stress and mood disorders with somatic diseases,^60^ and BDNF expression is regulated by stress.^61^ BDNF is a crucial mediator of neuronal events underlying learning and memory processes.^62^ Its expression is elevated in the hippocampus of animals that learn a spatial memory task, and reducing this expression results in spatial learning and memory deficits.^25^ Correspondingly, in the present study, we observed a significant reduction in hippocampal BDNF levels combined with spatial learning and memory deterioration after prolonged stress exposure, in agreement with previous reports.^63^,^64^

Current findings are consistent with the negative effect of prolonged stress on spatial learning and memory tested in the RAWM and BDNF levels in the hippocampus.^52^ Additionally, they shed light on the negative influence nine weeks of chronic stress on hippocampus-dependent memory processes. Herein, we will discuss possible mechanisms underlying the ameliorating effect of chronic *H. triquetrifolium* administration on stress-associated memory impairment.

### Stress, Spatial Acquisition and Retrieval

Long-term production of stress hormones challenges homeostatic mechanisms in the brain by over-activating stress systems that lead to negative morphological and functional changes in the brain,^30^,^65^ including hippocampal damage,^14^,^66^ and learning and memory deficits.^67^ The significance of the hippocampus in spatial learning and memory,^68^,^69^ and the negative influence of prolonged stress on hippocampal structure are well documented.^14^,^66^

The influence of chronic stress on spatial learning appears to be specific to the learning task used. The deterioration in spatial learning and memory performance exhibited by stressed rats in the present study is mostly related to the acquired within-day acquisition strategy used in which the platform location was changed daily. We deduced this because this protocol efficiently revealed stress-induced performance deficits in spatial learning and memory.^70–73^

Chronic stress had a negative impact on spatial learning, short-term (30 min) and 5 h memory retrieval. However, long-term (24 h) memory appeared to be preserved, with the performance of the chronically stressed group being comparable to that of the control group at this time point. This disparity is not surprising, since the stabilization and strengthening process for the initially labile memory goes through a complex process following the learning experience,^74^,^75^ occurs in stages,^76^,^77^ and is time-dependent.^77^ Post-encoding consolidation processes depend on the biological state of the tested animal, ie, whether it is active (awake) or inactive (asleep).^78^,^79^

Early studies on consolidation implicated the importance of sleep, and even classified memory according to biological state. For instance, according to the memory model introduced by Lewis (1979), formation of memory during the active and inactive states was suggested to be analogous to the formation of short-term (seconds to hours) and long-term (days to weeks) memories, respectively. This reflects the importance of sleep in long-term memory formation. Indeed, memory replay during sleep is crucial to long-term memory consolidation.^80^

Classically, consolidation was considered a process by which an initially labile memory trace was strengthened. Recently, sleep has been found to have a more significant role than merely stabilizing original memories. It involves a more complex process integrating the initially labile memory trace into established cortical memory networks,^81^,^82^ to extract meaning,^83^ and develop insight,^84^ leading to superior memory performance in later tests.

For declarative memory, accumulating evidence strongly implicates the importance of sleep in consolidation and in reactivation processes.^79^,^85^ Memory replay in sleep is crucial for the declarative form of memory consolidation.^86^ Hippocampal place cells, which encode the spatial context for memories, show enhanced firing during deep sleep after a session of spatial learning in the preceding waking periods.^87^,^88^ During subsequent sleep, improvement in hippocampus-mediated spatial memory occurs,^89^ and newly encoded spatial information gradually transits from short-term hippocampus dependence to long-term hippocampus independence by integrating into already-existing memory networks.^90^,^91^ Some studies suggest that the hippocampus-independent consolidation process develops up to 6 hours after the initial encoding.^74^ Others show that memory is enhanced in discrete stages of sleep via the “offline replay” of the newly encoded information, and integrated into larger associative networks in a process called “active system consolidation” during sleep.^75^,^92^

Current findings highlight the negative influence of chronic stress on hippocampus-based short-term (30 min) and 5 h memory retrieval. However, memory retrieval outside the hippocampus-based domain (24 h memory test) was re-normalized, which was probably due to the offline replay of the spatial information obtained in the preceding waking period during sleep. Together with the comparable performance of the sts and control groups in the 24 h memory test, it is suggested that an overnight improvement occurred in hippocampus-mediated spatial memory, thus normalizing memory in stressed animals. It also suggests that the hippocampus-independent long-term memory consolidation process remained intact despite the chronic elevation of stress hormones in the present study. In agreement with these results, BDNF protein expression was impaired only in the hippocampus and not in the other brain structures examined.

### Combined Effect of *H. triquetrifolium* on Learning and Memory in Chronically Stressed Rats

Chronic administration of *H. triquetrifolium* extract prevented the reduction in BDNF levels in chronically stressed rats, which could also explain behavioral findings from the current study. In addition to a BDNF protein-mediated mechanism, other postulated mechanisms could involve amelioration of oxidative stress status triggered by prolonged exposure to stress hormones by *H. triquetrifolium* extract. Alternatively, it could be due to the putative antidepressant activity of *H. triquetrifolium* extract, since it shares many biologically active components with *H. perforatum*, a herb widely used to relieve symptoms of depression.^36^

Chronic mild stress leads to a depression-like phenotype induced by a persistent state of oxidative stress.^19^ Depression is often associated with cognitive deficits and memory disturbances. A stress-triggered depressive phenotype also shows altered levels of BDNF protein.^93^ Furthermore, chronic stress impairs synaptic transmission, leading to downregulation of BDNF and resulting in sustained oxidative stress; this ultimately leads to a depression-like phenotype,^94^,^95^ and learning and memory deficits.^5^,^96^ This can be prevented by antioxidant treatment.^19^ *H. triquetrifolium* is an excellent free radical-scavenging herb,^47^,^48^ and the two compounds isolated in the present study, rutine and hypericin, are potent antioxidants.^97^,^98^ Rutin was previously described in *H. triquetrifolium*.^99^ The Antioxidant effect of rutin was already confirmed by a number of studies.^100–102^ It was shown to significantly elevate the activities of the antioxidant enzymes such as superoxide dismutase and catalase, and the levels of the antioxidant glutathione.^102^ Rutin effectively upregulated BDNF level resulting in alleviation of oxidative stress in hippocampal neurons.^102^ The high hypericin content of the *H. triquetrifolium* that is growing wild in Jordan was described by a previous study from our group.^103^ Hypericins are one of the main compounds in many species of the genus Hypericum. They are responsible for a wide variety of biological effects of Hypericum such as its antimicrobial, and antiviral effects.^104^ Importantly previous studies have reported stress-relieving and antidepressant effects for hypericin.^105^ The isolation of these compounds was part of a phytochemical study for quality purposes in the current project. Moreover, the amounts of the isolated compounds were not sufficient to design experiments to verify their action. Our future work will be covering this point.

At the molecular level, NMDA receptor dysregulation and BDNF downregulation in chronic stress are major contributors to the etiology of depression.^95^ The NMDA receptor sensitization is critical in the early phase of spatial acquisition.^106^ Even though hyperforin was not among isolated compounds in this study, *H. triquetrifolium* was found to have a high level of hyperforin.^107^ Hyperforin is an NMDA receptor antagonist,^108^ and is therefore potentially neuroprotective.^108^,^109^ When the antidepressant *H. perforatum* lacks hyperforin, it also lacks antidepressant-like properties, suggesting that hyperforin is the main active component responsible for the antidepressant properties of *H. perforatum*.^110^

Chronic stress and depression are closely associated and influence the hippocampus in a highly dynamic manner. Rats subjected to prolonged social stress show a broad spectrum of depression-related behaviors.^34^,^111^ Chronic stress and depression negatively influence memory formation, as well as cognitive and emotional processing. The hyperforin in *H. triquetrifolium* might attenuate depression- and anxiety-like behaviors in this rat model of chronic stress, thus, ameliorating the negative impact of chronic stress on memory processing.

Chronic stress that leads to a depression-like phenotype is also associated with reduced hippocampal BDNF levels.^112–114^ This effect was normalized by antidepressants,^112^,^115^ which also rescue stress hormone-triggered impairments in spatial memory.^116^ Therefore, in addition to the powerful antioxidant properties of hyperforin in *H. triquetrifolium*, we propose that its NMDA antagonist action prevents BDNF downregulation and improves depression- and anxiety-like behaviors. This could prevent chronic stress-induced disruption to learning and memory processes.

To our best knowledge, this is the ﬁrst study to show that methanolic extract of *H. triquetrifolium* ameliorates stress-associated hippocampal-dependent memory deficits via a mechanism involving BDNF in the hippocampus. In humans, the vast majority of research has focused on St. John’s wort (*Hypericum perforatum L*.) extract, a closely related species to *H. triquetrifolium. H. perforatum*, that used for centuries to clinically treat a number of common ailments, eg, neuralgia, sleep pattern disturbances, wound healing and hemorrhoids.^117^,^118^ However, it gained popularity for its use to relieve symptoms of mild to moderate depression.^119^ Both *H. triquetrifolium and H. perforatum* belong to *Hypericaceae* family and share many of biologically active compounds.^46^

Multiple doses of *Hypericum perforatum L* neither enhanced nor deteriorated cognitive functions in healthy volunteers when tested in various behavioral paradigms such as; choice reaction, psychomotor coordination, short-term memory and responsiveness to distractive stimuli.^120^ This is in line with the current study’s findings, which revealed that the restorative effect on memory function of *H. triquetrifolium* is more prominent in deficit models but not in non-deficit models.

Further research with more behavioural dimensions is needed to test the hypothesis that *H. triquetrifolium* extract can rescue hippocampal function from the negative effect of chronically elevated stress hormones. Indeed, a more comprehensive behavioural profile is needed to extract emotional, attention and anxiety and reveal the impact of chronic administration of *H. triquetrifolium* extract. Moreover, investigations of the pharmacological activities of *H. triquetrifolium* on other forms of learning and memory impairment in psychiatric diseases are highly recommended. One limitation of this study is that we did not examine oxidative stress biomarkers such as reduced/oxidized glutathione ratio, glutathione peroxidase and catalase activity, and oxidized glutathione levels. This would be worth doing in a future study since *H. triquetrifolium* extract has already been shown to have excellent antioxidant properties.^47^ Besides the amelioration effect of oxidative stress by rutin when tested in differentiated neuronal cells.^101^ Another limitation of the current study is the test of a single dose of *H. triquetrifolium* extract, and the need for positive control as part of the study. Future work should also investigate the effect of multiple doses of *H. triquetrifolium* extract on hippocampus-dependent learning and memory with incorporation of positive controls as needed.

## Conclusion

The data from this study indicate that chronic psychosocial stress can impair hippocampus-dependent memory processing. Chronic administration of *H. triquetrifolium* extract attenuated the negative impact on spatial learning and retrieval and normalized BDNF levels in the hippocampus. However, long-term treatment with *H. triquetrifolium* neither improved nor reduced spatial ability when tested in the aversively motivated water maze task. Thus, the restorative effect on memory function of *H. triquetrifolium* could be more prominent in deficit models than in non-deficit models, which is in line with the positive impact of chronic *H. triquetrifolium* on memory function and BDNF levels in chronically stressed, but not unstressed, rats. These properties of *H. triquetrifolium* extract could be of interest for future intervention studies.
